# Molecular screening for Lynch syndrome in early-onset colorectal cancer in Northern Tanzania

**DOI:** 10.3332/ecancer.2026.ed136

**Published:** 2026-07-16

**Authors:** Alex Mremi, Ayesiga Herman, Furaha Serventi, Athanasia Maro, Daniel Mbwambo, Arjen R Mensenkamp, Janneke H M Schuurs-Hoeijmakers, Ben C J Hamel

**Affiliations:** 1 Department of Pathology, Kilimanjaro Christin Medical Centre, PO Box 3010, Moshi, Tanzania; 2 School of Medicine, KCMC University, PO Box 2240, Moshi, Tanzania; 3 Kilimanjaro Clinical Research Institute, PO Box 2236, Moshi, Tanzania; 4 Department of Surgery, Kilimanjaro Christian Medical Centre, PO Box 3010, Moshi, Tanzaia; 5 Cancer Care Centre, Kilimanjaro Christian Medical Centre, PO Box 3010, Moshi, Tanzania; 6 Department of Human Genetics, Radboud university medical center, Box 6526 GA, Nijmegen, The Netherlands; 7 Department of Pathology, Radboud university medical center, Box 6526 GA, Nijmegen, The Netherlands; a https://orcid.org/0000-0001-7226-0168

**Keywords:** colorectal neoplasms, Lynch syndrome, microsatellite instability, DNA mismatch repair, genetic testing, Tanzania

## Abstract

Colorectal cancer (CRC) incidence is increasing in sub-Saharan Africa, with a growing burden of early-onset disease and advanced-stage presentation. The contribution of Lynch syndrome (LS), the most common hereditary cause of CRC, remains poorly defined in this region. We conducted a retrospective pilot study among patients aged ≤50 years diagnosed with CRC in northern Tanzania to determine the prevalence of LS. Tumour samples were initially screened for mismatch repair (MMR) deficiency using immunohistochemistry and microsatellite instability (MSI) testing. Cases demonstrating MMR protein loss underwent MLH1 promoter hypermethylation analysis, and those without hypermethylation were subsequently selected for germline sequencing of MMR genes. Among 47 patients, six (12.8%) had MSI–high tumours and three (6.4%) carried pathogenic germline variants consistent with LS. The observed prevalence is comparable to reports from high-income countries and other African cohorts. These findings demonstrate that LS contributes meaningfully to early-onset CRC in Tanzania and support the need to expand molecular diagnostics and hereditary cancer screening in resource-limited settings.

## Introduction

Colorectal cancer (CRC) incidence is rising across sub-Saharan Africa (SSA), with increasing reports of early-onset disease and advanced-stage presentation [1]. In Tanzania and other SSA countries, recent reports show a growing burden of CRC among younger adults, with up to 90% of patients presenting with stage III or IV disease, contributing to poor survival outcomes [2–6]. Despite this shifting epidemiology, the contribution of hereditary cancer syndromes – particularly Lynch syndrome (LS) – remains largely unexplored.

LS, an autosomal dominant disorder caused by pathogenic germline variants in the mismatch repair (MMR) genes *MLH1, MSH2, MSH6* or *PMS2*, or *EPCAM* deletions – is the most common hereditary cause of CRC. LS accounts for 2%–3% of all CRC in Western populations and an estimated 4%–13% of cancers before age 50 [7–9]. Individuals with LS typically develop CRC at a younger age and benefit from targeted surveillance, risk-reducing strategies and cascade testing of at-risk relatives. Despite this, data on LS in SSA remain scarce, and to the best of our knowledge, no previous studies have evaluated the prevalence of LS in Tanzania. Limited access to MMR immunohistochemistry (IHC), microsatellite instability (MSI) testing and germline sequencing further constrains the ability to detect LS in the region.

To address this knowledge gap, we conducted a pilot study to determine the prevalence of LS among patients aged ≤50 years diagnosed with CRC in northern Tanzania. Using a combination of IHC, MSI testing and germline analysis, we aimed to characterize the frequency of MMR deficiency and identify pathogenic variants indicative of LS in this understudied population. These data represent an essential first step toward integrating genetic risk assessment into CRC care in Tanzania.

## Methods

### Study design and patient selection

We performed a retrospective pilot study of CRC patients aged ≤50 years who underwent surgical resection or biopsy at a tertiary referral centre in northern Tanzania. All consecutive patients meeting the age criterion with available formalin-fixed, paraffin-embedded (FFPE) tissue blocks were eligible. Specimens collected between 2017 and 2022 were reviewed. Cases were excluded if the tissue was insufficient for analysis or severely degraded.

Clinical and pathological information, including demographics, presenting symptoms, tumour location, histologic subtype, tumour grade and stage – were extracted from patient records. Family history data were not systematically recorded (Table 1).

### IHC and MLH1 hypermethylation analysis

FFPE tumour blocks were shipped under controlled conditions to the pathology laboratory at Radboud university medical center (Nijmegen, The Netherlands) for diagnostic consultation. Histopathologic review confirmed primary malignancy of the colon or rectum in all selected cases. IHC was performed for the four MMR proteins (*MLH1, MSH2, MSH6, PMS2*) following standard protocols. Complete loss of nuclear staining in tumour cells with intact internal controls (e.g., lymphocytes, stromal cells) was interpreted as MMR-deficient. Tumours were categorized according to the pattern of protein loss: MLH1-deficient (nuclear loss of MLH1 and PMS2), PMS2-deficient (isolated loss of PMS2), MSH2-deficient (loss of MSH2 and MSH6) or MSH6-deficient (isolated loss of MSH6).

MLH1 promoter hypermethylation was detected using methylation-specific multiplex ligation-dependent probe amplification (MRC Holland ME011 kit) according to routine diagnostic procedures.

### Molecular (germline and somatic) analysis

Genomic DNA was extracted from paired normal and tumour FFPE tissue for germline and somatic mutation analysis of the MMR genes, including next-generation sequencing (NGS) MSI analysis. This analysis was restricted to the cases with MMR loss without hypermethylation of the MLH1 promoter, using single-molecule molecular inversion probe–based NGS, as described previously [10–12]. Variant interpretation followed American College of Medical Genetics and Genomics guidelines [13]. Loss of heterozygosity (LOH) and secondary somatic events were documented when present.

### Statistics

Descriptive statistics were used to summarize clinico-pathological characteristics. Continuous variables were summarized as medians with interquartile ranges, and categorical variables as frequencies and percentages. Comparisons were made between MSI and non-MSI tumours. Statistical analyses were performed in STATA 17 (StataCorp, College Station, TX, USA). A two-sided *p*-value of <0.05 was considered statistically significant.

### Ethical considerations

All analyses were performed on diagnostic samples submitted as part of routine clinical evaluation. After completion of diagnostic reporting, de-identified results were reviewed retrospectively. No identifiable patient data were accessed. According to institutional policies at the participating centres, this study qualifies as a retrospective service evaluation, as it involved the secondary use of existing diagnostic samples and routinely collected clinical data without any direct patient contact or intervention. Under this framework, such analyses are not classified as human subjects’ research requiring formal approval from an Institutional Review Board or equivalent ethics committee.

## Results

Between 2017 and 2022, a total of 321 CRC cases were recorded at our centre, from which the early-onset study cohort was selected. Most clinico-pathological characteristics did not differ significantly between MSI-high (MSI-H) and non-MSI tumours (Table 1). Fisher’s exact test confirmed no statistically significant associations between MSI status and these variables; however, non-significant trends were observed for longer symptom duration before diagnosis (*p* = 0.07) and a higher frequency of abdominal pain or distension in MSI-H tumours (*p* = 0.09), likely reflecting limited statistical power.

Among 47 patients aged ≤50 years, 6 (12.8%) had MSI-H tumours. Of these, three patients (6.4% of the total cohort) harbored pathogenic germline variants in MMR genes (*MSH2* or *MSH6*) (Table 2). A high proportion of patients presented with advanced clinical features, including obstructive symptoms (63.8%), emergency presentation (66%) and advanced-stage disease (72.3%) (Table 1).

Molecular analysis identified pathogenic germline variants in *MSH2* and *MSH6*. In addition, several MSI-H tumours demonstrated somatic biallelic inactivation of MMR genes, consistent with Lynch-like tumour profiles. Regarding sample integrity, most tumour specimens were adequate for molecular analysis; however, one case yielded unreliable MSI results due to suboptimal tissue quality.

## Discussion

This study provides the first characterization of LS among early-onset CRC patients in Tanzania, demonstrating a prevalence of 6.4%. This estimate is comparable to reported rates in early-onset CRC cohorts from high-income settings (4%–13%) and aligns with findings from other SSA populations, including Congo-Brazzaville (5.6%) and Zimbabwe (7.7%) [14, 15]. Differences across studies likely reflect small sample sizes, variations in patient selection criteria, access to germline testing and underlying genetic heterogeneity. Importantly, given the enrichment of hereditary syndromes in early-onset CRC, comparisons should be restricted to similarly defined cohorts.

The absence of distinct clinico-pathological differences between MSI-H and non-MSI tumours is consistent with prior observations that early-onset CRC often presents with nonspecific symptoms, irrespective of genetic etiology [7, 8]. Although MSI-H tumours were more frequently associated with abdominal symptoms and longer symptom duration, these features lack sufficient specificity to guide the identification of hereditary cancer risk in clinical practice.

The high rates of obstructive symptoms, emergency presentation and advanced-stage disease reflect persistent delays in CRC diagnosis in SSA [16–19]. In this context, identifying LS has particular clinical significance, as cascade testing and targeted surveillance of at-risk relatives can substantially reduce morbidity and mortality.

The detection of germline variants in *MSH2* (two patients, onset CRC 40 and 44 years) and *MSH6* (one patient, onset 47 years) is consistent with global trends showing MLH1 and MSH2 as the most prevalent causes of LS in early-onset CRC, followed by MSH6 [7]. While *PMS2* shows lower penetrance and a later onset. The identification of somatic biallelic inactivation events in several MSI-H tumours highlights the importance of distinguishing LS from ‘Lynch-like’ tumours, which arise from somatic MMR gene alterations rather than germline variants. Clarifying this distinction is crucial for determining which families require genetic counseling and cascade testing.

Implementation of routine MMR IHC, MSI testing and selective germline sequencing has the potential to significantly improve CRC care in Tanzania. However, major barriers remain, including limited access to molecular diagnostics, shortages of trained genetic counselors and financial constraints [10, 11]. Addressing these challenges will require coordinated national strategies, investment in laboratory capacity and workforce development in cancer genetics.

While our study primarily focused on molecular characterization using MMR IHC and MSI testing, we recognize that routine hematoxylin and eosin evaluation can provide useful initial clues to underlying MMR deficiency, especially in resource-limited settings. In this cohort, histopathological review was limited to tumour type, grade and histologic subtype, and we did not systematically assess features commonly associated with MMR deficiency, such as tumour-infiltrating lymphocytes, mucinous or medullary differentiation or Crohn-like lymphoid reaction. In settings where access to molecular testing is limited, careful attention to these morphological features could help identify cases that warrant further testing, allowing for more efficient use of available resources. Future work in this context should aim to incorporate standardized assessment of these features to better understand their practical role in guiding cost-effective LS screening strategies.

This study has important limitations: from a systems perspective, variability in tissue quality highlights a key operational constraint, underscoring the need to strengthen pathology infrastructure – including standardized tissue handling, storage and quality assurance – to support reliable molecular diagnostics. In addition, the relatively small sample size and single-centre design may limit the generalizability of the findings; incomplete documentation of family history precluded application of established clinical criteria such as the Amsterdam or Bethesda guidelines for LS identification; germline testing was restricted to selected cases with MMR deficiency, potentially leading to underestimation of LS prevalence; and the retrospective study design, together with occasional suboptimal tissue quality, may have affected the completeness and reliability of molecular analyses.

## Conclusion

Our findings demonstrate that LS is an important contributor to early-onset CRC in Tanzania. Expanding diagnostic capacity for MMR deficiency testing, including MSI analysis and MLH1 hypermethylation testing, followed by targeted germline evaluation, is critical. Establishing systematic LS screening programs and enabling cascade testing could substantially reduce the burden of advanced CRC in Tanzania and across the region.

## List of abbreviations

CRC, Colorectal cancer; EPCAM, Epithelial cell adhesion molecule; FFPE, Formalin-fixed, paraffin-embedded; HM-MLH1, MLH1 promoter hypermethylation; IHC, Immunohistochemistry; LOH, Loss of heterozygosity; LS, Lynch syndrome; MLH1, MutL homolog 1 (MMR gene); MLPA, Multiplex ligation-dependent probe amplification; MMR, Mismatch repair; MSH2, MutS homolog 2 (MMR gene); MSH6, MutS homolog 6 (MMR gene); MSI, Microsatellite instability; MSI-H, Microsatellite instability–high; NGS, Next-generation sequencing; PMS2, Postmeiotic segregation increased 2 (MMR gene); SSA, Sub-Saharan Africa; VUS, Variant of unknown significance; n/a – Not applicable.

## Conflicts of interest

All authors declared no conflicts of interest exist.

## Funding

Alex Mremi and Athanasia Maro gratefully acknowledge funding from the Science for Africa Foundation with support from Wellcome Trust and the UK Foreign, Commonwealth & Development Office and part of the EDCPT2 programme supported by the European Union for supporting.

## Figures and Tables

**Table 1.** Socio-demographic and clinico-pathological characteristics of study subjects, N = 47.

Variable	N (%)	MSI tumors (LS cases)	Non-MSI tumors	p-value
Age (Years)				0.68
≤40	19 (40.4%)	2 (1)	17	
41–50	28 (59.6%)	4 (2)	24	
Sex				0.38
Male	27 (57.4%)	2 (1)	25	
Female	20 (42.6%)	4 (2)	16	
Year of diagnosis				0.41
2017–2019	25 (53.2%)	2 (2)	23	
2020–2022	22 (46.8%)	1 (1)	21	
Presentation mode				1.00
Elective	16 (34.0%)	2 (1)	14	
Emergency	31 (66.0%)	4 (2)	27	
Symptoms				0.09
Rectal bleeding	26 (55.3%)	3 (2)	23	
Constipation	38 (80.9%)	3 (1)	35	
Intestinal obstruction	30 (63.8%)	2 (2)	28	
Weight loss	27 (57.4%)	2 (1)	25	
Anemia	20 (42.6%)	2 (1)	18	
Mucoid stool	10 (21.3%)	1 (1)	9	
Abdominal distension/pain	18 (38.3%)	4 (2)	4	
Symptom duration				0.07
0–4 weeks	11 (23.4%)	0	11	
5–12 weeks	17 (36.2%)	1 (1)	16	
13+ weeks	19 (40.4%)	5 (2)	14	
Tumor location				0.38
Right colon	16 (34.0%)	2	14	
Left colon	17 (29.8%)	2 (1)	15	
Rectum	14 (29.8%)	2 (2)	12	
Histopathology				0.33
Adenocarcinoma	34 (72.3%)	4 (2)	30	
Mucinous carcinoma	11 (23.4%)	1 (1)	10	
Signet ring cell	2 (4.2%)	1	1	
Tumor grade				1.00
Well/moderate differentiated	28 (59.6%)	4 (2)	24	
Poorly differentiated	19 (40.4%)	2 (1)	17	
T stage				0.16
T1–T2	13 (27.6%)	0	13	
T3–T4	34 (72.4%)	6 (3)	28	
MSI: microsatellite instability; LS: Lynch syndrome with pathogenic germline variant				

**Table 2.** Results of molecular analyses in MSI-H cases.

Case	Age	MSI status	IHC	HM-MLH1	Variants normal tissue	Variants tumor tissue	Second variant tumor tissue	Conclusion/remarks
1	40	MSI-H	MSH2/MSH6 deficient	n/a	MSH2 c.425C>A p.(Ser142*) 42% 24 reads	MSH2 c.425C>A p.(Ser142*) 91% 22 reads, LOH		Germline MSH2
2	45	MSI-H	MSH2/MSH6 deficient	n/a	No pathogenic variant detected	MSH2 c.1165C>T p.(Arg389*) 72%, LOH	MSH6 c.3261del p.(Phe1088fs) 13% MSI variant	Somatic MSH2
3	47	MSI-H	MSH6 deficient	n/a	MSH6 c.3261dup p.(Phe1088fs) 50%	MSH6 c.3261dup p.(Phe1088fs) 68%, LOH		Germline MSH6
4	44	MSI-H	MSH2/MSH6 deficient	n/a	MSH2 c.425C>A p.(Ser142*) 53%	MSH2 c.425C>A p.(Ser142*) 46% and VUS c.1896_1898del p.(Ile633del) 33%	MSH6 c.3261del p.(Phe1088fs) 28% MSI variant	Germline MSH2
5	45	MSI-H	MLH1/PMS2 deficient	absent	No pathogenic variant detected	MLH1: c.617_620dup p.(Pro208fs) and LOH		Somatic MLH1
6	25	MSI-H	MLH1/PMS2 deficient	absent	No pathogenic variant detected	MLH1: c.813del p.(Leu272*) + LOH		Somatic MLH1
MSI-H: microsatellite instability-high; n/a: not applicable; LOH: loss of heterozygosity; VUS: variant of unknown significance								
